# Synchronous intracranial arteriovenous malformation and papillary glioneuronal tumour: hypothesis or reality?

**DOI:** 10.1007/s00381-024-06621-3

**Published:** 2024-09-18

**Authors:** Michael Stuart, Adam Burnett, Thomas Robertson, Annabelle Harbison, Liam Coulthard, Robert Campbell

**Affiliations:** 1 Department of Neurosurgery, Queensland Children’s Hospital, 501 Stanley St, South Brisbane, QLD 4101 Australia; 2 Department of Neurosurgery, Princess Alexandra Hospital, Woolloongabba, QLD Australia; 3 Pathology Queensland, Royal Brisbane and Women’s Hospital, Herston, QLD Australia; 4 School of Biomedical Sciences, The University of Queensland, St Lucia, QLD Australia; 5 School of Medicine, The University of Queensland, St Lucia, QLD Australia

**Keywords:** Glioma, Cerebrovascular, Paediatric, Angioglioma

## Abstract

Brain arteriovenous malformations (AVM) rarely occur with spatial and/or temporal co-localisation to intracranial neoplasms. Most prior reports describe this association with high-grade gliomas; however, reports of a co-occurrence with low grade gliomas are very rare. It is unclear whether such cases represent a true co-occurrence of separate pathologies or simply an unusually vascular phenotype of the neoplasm. Most such reports pre-date the era of molecularly defined gliomas. We present the first report of the spatial and temporal co-occurrence of an intracranial arteriovenous malformation traversing and within a papillary glioneuronal tumour, molecularly defined by the presence of *SLC44A1::PRKCA* fusion. This case was successfully managed by resection of both lesions adhering to the principles of AVM surgery. It is possible these exceptionally rare co-occurrences may have common underlying molecular drivers relating to the mitogen activated protein kinase (MAPK) pathway.

## Introduction

The comorbidity of arteriovenous malformations (AVMs) with primary intracranial neoplasms is relatively well documented, but poorly understood [1–7]. The relationship between the two can be categorised in terms of their spatial and temporal co-occurrence, for example the occurrence of glioblastoma adjacent to an AVM nidus years following stereotaxic radiosurgery demonstrates spatial co-occurrence but temporal dissemination and holds little mystery regarding the mechanisms of that relationship. Likewise contralateral AVM and gliomas discovered simultaneously (spatially dissociated but temporally related) could be readily explained as coincidental. Those lesions which generate most interest in terms of both the underlying pathophysiology and complexities of their management are those with both spatial and temporal colocalization. There is controversy within the existing literature regarding whether those lesions represent either a co-occurrence of separate pathologies (either by chance or some common underlying mechanism), a single pathology (e.g. an unusually vascular glioblastoma phenotype), or a unique entity—occasionally termed ‘angioglioma’ [1–7]. Previous reports of spatially and temporally co-occurring AVM and glioma predominantly consist of high-grade gliomas such as glioblastoma, with very scarce reports of co-occurring AVM and low grade gliomas [1–7]. In total, a recent review identified only 41 cases (half confirmed with angiography) previously reported in the literature, of which eight were low grade gliomas [1]. These lesions are exceptionally rare and most reports pre-date the incorporation of molecular diagnosis in the 2021 World Health Organization classification [8]. We present the first report of the spatial and temporal co-localisation of a large AVM with a molecularly defined papillary glioneuronal tumour (PGNT), whose defining molecular pathway alteration has also been implicated in the pathogenesis of AVMs.

## Case

A 10-year-old girl presented to a local optometrist with six months of headaches and nausea. No previous clinical episodes to suggest seizures or intracranial haemorrhage were noted.

**Fig. 1:**
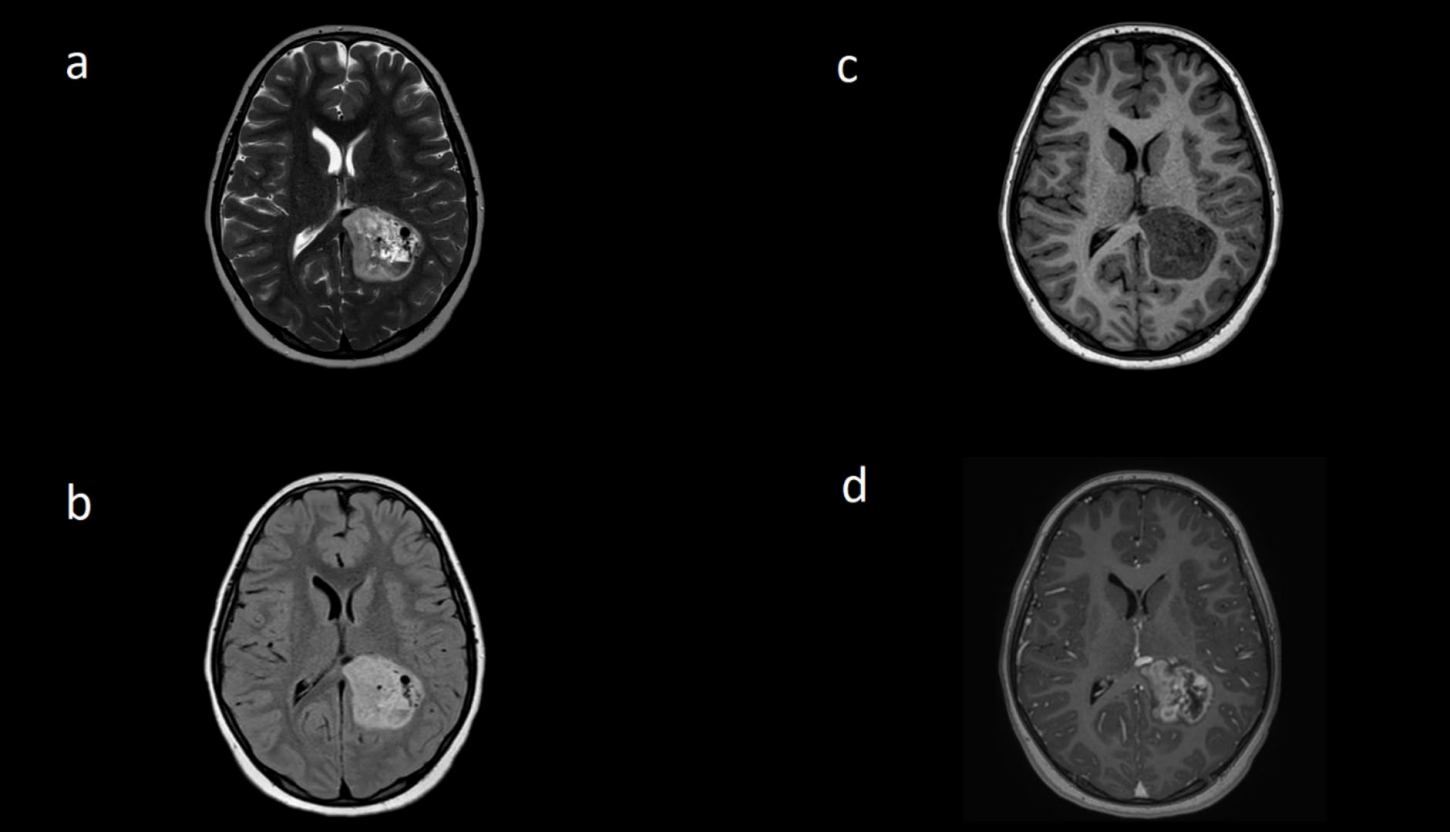
Preoperative axial MRI sequences: **a** T2, **b** FLAIR, **c** T1 and **d** T1 + gadolinium

**Fig. 2:**
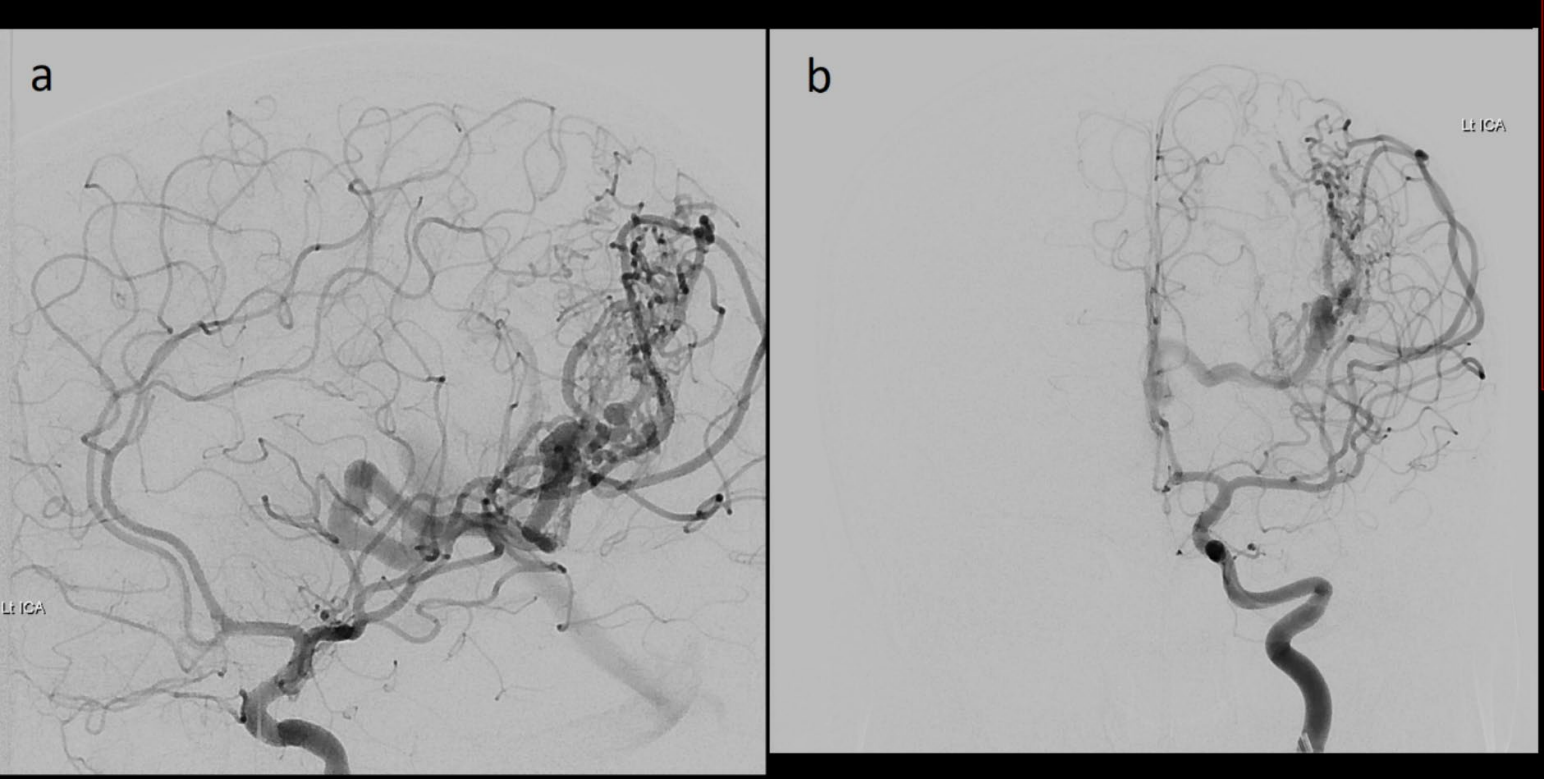
Preoperative DSA in late arterial phase: **a** antero-posterior and **b** lateral views

Magnetic resonance imaging (MRI) demonstrated a left parietal T1 hypointense, T2 hyperintense lesion with peripheral contrast enhancement but without diffusion restriction. Susceptibility-weighted imaging demonstrated low signal throughout the lesion suspicious for previous haemorrhage (Fig. 1). The lesion was traversed by large flow voids both within and without the T2 hyperintensity and a conspicuous draining vein. Subsequent digital subtraction angiogram (DSA) confirmed the presence of a high-flow arteriovenous malformation (49 × 44 × 32 mm) of Spetzler-Martin Grade 3 with feeding vessels from the middle cerebral, posterior cerebral and anterior choroidal arteries and deep venous drainage to the internal cerebral vein (Fig. 2). The patient underwent resection of the lesion in accordance with the principles of AVM surgery—ligation of feeders, followed by circumnavigation and division of the deep draining vein as the final step. Intraoperative frozen section was suggestive of a neoplasm with papillary architecture in the biopsied T2 hyperintensity, which was therefore resected in its entirety. Postoperative imaging confirmed gross total resection of both lesions, and the patient had an uncomplicated postoperative course without neurological deficits. Three years of subsequent surveillance MRI confirm no recurrence.

## Pathology

**Fig. 3:**
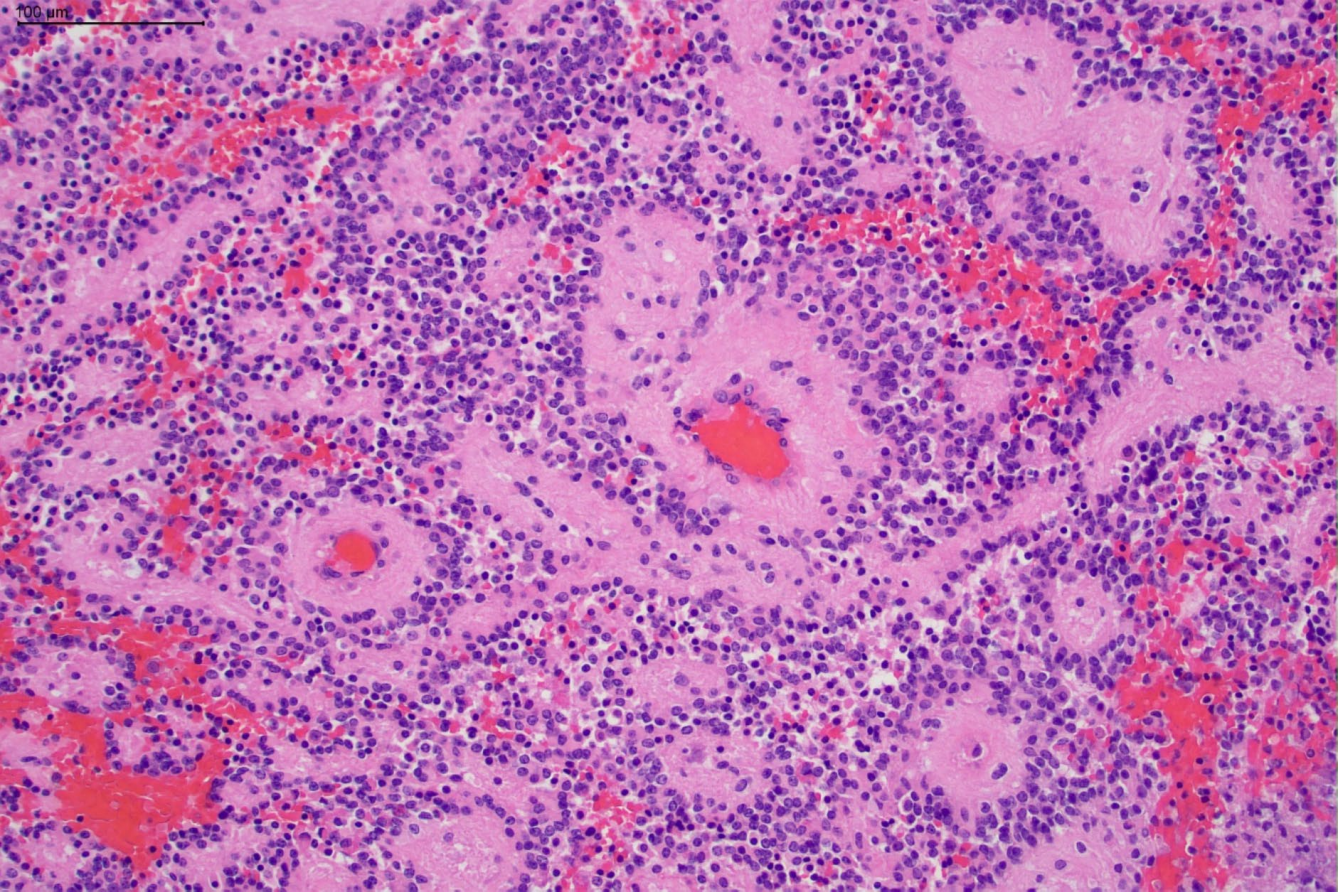
Hematoxylin and eosin–stained photomicrographs: High power view demonstrating the PGNT with a solid papillary architecture and perivascular pseudorosettes (scale bar 100 μm)

**Fig. 4:**
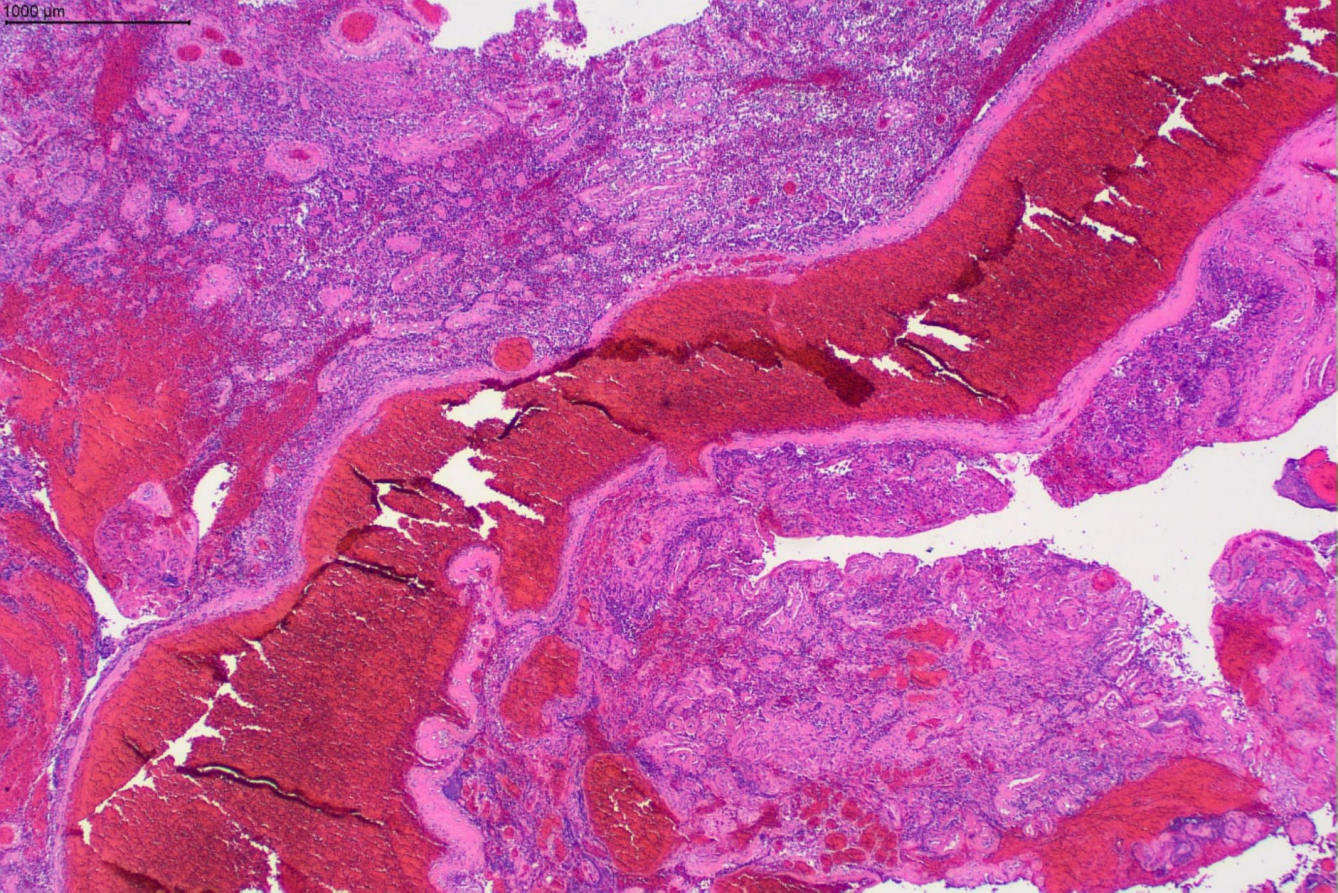
Hematoxylin and eosin–stained photomicrographs: Low power view demonstrating large fibrotic vasculature with an irregular calibre compatible with an arteriovenous malformation associated with the tumour (scale bar 1 mm)

Histopathology confirmed the presence of a primary neoplasm composed of relatively monomorphous small epithelioid cells with perivascular pseudorosette formation and areas of prominent fibrotic vasculature without endothelial proliferation (Figs. 3, 4). The lesion was positive for the immunohistochemical glial markers Glial Fibrillary Acidic Protein (GFAP), Olig2 and SOX10 but also showed focal immunoreactivity for the neuronal markers synaptophysin and NeuN. The lesion was isocitrate dehydrogenase (IDH) wildtype. MN1 was not altered. The Ki67 index was < 5%. Subsequent whole genome sequencing detected a *SLC44A1::PRKCA* fusion characteristic of papillary glioneuronal tumour which provided the final diagnosis. The presence of large fibrotic vessels traversing this lesion is not a previously described phenotype. There are previous reports of prominent vascularity within these lesions; however, these more typically consist of smaller fibrovascular cores with the associated papillary architecture.^8^

## Discussion

Papillary glioneuronal tumour is a rare neoplasm classified as WHO Grade 1 [8, 9]. The prognosis of PGNT is favourable and there are few reports of recurrence following gross total resection [8]. Histologically and immunohistochemically, PGNT is not readily differentiated from other glioneuronal tumours; however, *SLC44A1::PRKCA* fusion appears to be unique to PGNT and is now regarded as its defining molecular feature [8, 9].

PRKCA encodes for protein kinase C alpha (PKCα) and the fusion of this gene creates a constitutively active serine/threonine kinase fusion protein [9]. PKCα plays a role in cell differentiation and proliferation and elevated PKCα activity has been implicated in oncogenesis [10]. PKCα is involved in regulating mitogen activated protein kinase (MAPK) signalling through its interactions with RAF-1 [9]. The MAPK pathway is widely implicated in the genesis of many neoplastic lesion including gliomas, but also in arteriovenous malformations [11, 12]. Recent studies have implicated activation of the RAS/MAPK signalling pathway as a point of commonality between syndromic and sporadic AVMs [13]. In high-grade gliomas, peri-necrotic hypoxia and increased secretion of vascular endothelial growth factor (VEGF) are well-described drivers of neovascularisation, and again have been implicated in the recruitment of feeders to AVMs [3, 6, 7].

Whilst there are plausible common drivers, there does not appear to be a large burden of unrecognised neoplasms associated with otherwise typical AVMs. Lombardi and colleagues reviewed 1034 AVM specimens and found no instances of glioma within the specimens [2]. Another series found only 2 cases of simultaneous glioma and AVM from a cohort of 2277 patients with primary brain tumours and 222 patients with AVMs [4].

The single case reported here should be viewed as hypothesis generating and our favoured interpretation is a co-occurrence of AVM and PGNT, perhaps on the basis of a common underlying molecular pathway. However, as with all previous reports, it is not possible to totally exclude; however, this may be simply an unusually vascular phenotype of this rare tumour.

Perhaps, the key clinical lesson to be derived from this case is that in the co-occurrence of AVM and neoplasm, a surgical strategy adhering to the principles of AVM surgery is prudent.

## Conclusion

We present the first report of the co-occurrence of an AVM within a PGNT, which may be of relevance to the future understanding of common molecular drivers between intracranial AVMs and neoplasms.

## Data Availability

No datasets were generated or analysed during the current study.

## References

[1] Tunthanathip T, Kanjanapradit K (2020) Glioblastoma multiforme associated with arteriovenous malformation: a case report and literature review. Ann Indian Acad Neurol Jan-Feb 23(1):103–106. 10.4103/aian.AIAN_219_18 10.4103/aian.AIAN_219_18 PMC7001429 32055129

[2] Lombardi D, Scheithauer BW, Piepgras D, Meyer FB, Forbes GS (1991) “Angioglioma” and the arteriovenous malformation-glioma association. J Neurosurg 75(4):589–666. 10.3171/jns.1991.75.4.0589 1885977 10.3171/jns.1991.75.4.0589

[3] Lohkamp LN, Strong C, Rojas R, Anderson M, Laviv Y, Kasper EM (2016) Hypervascular glioblastoma multiforme or arteriovenous malformation associated Glioma? A diagnostic and therapeutic challenge: A case report. Surg Neurol Int 7(Suppl 37):S883–S888. 10.4103/2152-7806.194506 27999714 10.4103/2152-7806.194506 PMC5154202

[4] Licata C, Pasqualin A, Freschini A, Barone G, Da Pian R (1986) Management of associated primary cerebral neoplasms and vascular malformations: 2 Intracranial arterio-venous malformations. Acta Neurochir (Wien) 83(1–2):38–46. 10.1007/BF01420506 3799248 10.1007/BF01420506

[5] Li LR, Chen SY, Yang MY, Cheng WY, Shen CC (2023) A rare triploid involving the coexistence of glioblastoma multiforme, arteriovenous malformation and intracranial aneurysm: illustrative case and literature review. Medicina (Kaunas) 59(2):331. 10.3390/medicina59020331 36837531 10.3390/medicina59020331 PMC9966677

[6] Gmeiner M, Sonnberger M, Wurm G, Weis S (2013) Glioblastoma with the appearance of arteriovenous malformation: pitfalls in diagnosis. Clin Neurol Neurosurg 115(5):501–506. 10.1016/j.clineuro.2012.12.009 23290419 10.1016/j.clineuro.2012.12.009

[7] Khanna A, Venteicher AS, Walcott BP, et al (2013) Glioblastoma mimicking an arteriovenous malformation. Clinical case study. Front Neurol 4. 10.3389/fneur.2013.00144 10.3389/fneur.2013.00144 PMC3786388 24137154

[8] WHO Classification of Tumours Editorial Board (2021) World health organization classificaiton of tumours, 5th edn, vol 6. Central nervous system tumours, pp 130–133

[9] Pages M, Lacroix L, Tauziede-Espariat A et al (2015) Papillary glioneuronal tumors: histological and molecular characteristics and diagnostic value of SLC44A1-PRKCA fusion. Acta Neuropathol Commun 3:85. 10.1186/s40478-015-0264-5 26671581 10.1186/s40478-015-0264-5 PMC4681033

[10] Baltuch GH, Dooley NP, Rostworowski KM, Villemure JG, Yong VW (1995) Protein kinase C isoform alpha overexpression in C6 glioma cells and its role in cell proliferation. J Neurooncol 24(3):241–250. 10.1007/BF01052840 7595754 10.1007/BF01052840

[11] Lawton MT, Rutledge WC, Kim H et al (2015) Brain arteriovenous malformations. Nat Rev Dis Primers 1:15008. 10.1038/nrdp.2015.8 27188382 10.1038/nrdp.2015.8

[12] Walcott BP, Winkler EA, Rouleau GA, Lawton MT (2016) Molecular, cellular, and genetic determinants of sporadic brain arteriovenous malformations. Neurosurgery 63(1):37–42. 10.1227/NEU.0000000000001300 27399362 10.1227/NEU.0000000000001300 PMC4941635

[13] Peterson K, Coffman S, Zehri A, Anzalone A, Xiang Z, Wolfe S (2021) Somatic mosaicism in the pathogenesis of de novo cerebral arteriovenous malformations: a paradigm shift implicating the RAS-MAPK signaling cascade. Cerebrovasc Dis 50(2):231–238. 10.1159/000512800 33556951 10.1159/000512800

